# Evaluation of *C-C Motif Chemokine Receptor 5* (*CCR5*) as a Sample Adequacy Control in HPV Molecular Diagnostics

**DOI:** 10.3390/diagnostics14192194

**Published:** 2024-09-30

**Authors:** Ruth C. Njoku, Marianna Martinelli, Chiara Giubbi, Sofia De Marco, Barbara Torsello, Morena d’Avenia, Manuela Sironi, Cristina Bianchi, Clementina E. Cocuzza

**Affiliations:** 1 Department of Biomedical Sciences, University of Sassari, 07100 Sassari, Italy; r.njoku@phd.uniss.it; 2 School of Medicine and Surgery, University of Milano-Bicocca, 20126 Milan, Italy; chiara.giubbi@unimib.it (C.G.); sofia.demarco@unimib.it (S.D.M.); barbara.torsello@unimib.it (B.T.); manuela.sironi@unimib.it (M.S.); cristina.bianchi@unimib.it (C.B.); clementina.cocuzza@unimib.it (C.E.C.); 3 UOSVD of Cytopathology and Screening, Department of Laboratory Medicines, Ospedale di Venere, Asl Bari, 70132 Bari, Italy; morena.davenia@asl.bari.it

**Keywords:** *C-C Motif Chemokine Receptor 5* (*CCR5*), Human Papillomavirus (HPV), sample adequacy control (SAC), OncoPredict HPV Quality Control (QC) assay

## Abstract

**Background:** Reliable Human Papillomavirus (HPV) testing and genotyping are essential for quality assurance in HPV-based primary screening, disease management and for monitoring the impact of HPV vaccination. The clinical validation of HPV molecular diagnostic assays has significantly contributed to these objectives; however, little emphasis has been placed on assuring sample quality. This study aimed to evaluate the accuracy of sample cellularity assessment using the *C-C Motif Chemokine Receptor 5* (*CCR5*) gene target as a marker of sample adequacy in molecular diagnostics. **Methods:** Jurkat cell line samples were counted using both a Thoma cell-counting chamber and Fluorescence-Activated Cell Sorting (FACS). Jurkat cell line samples at three different concentrations were subsequently evaluated using the OncoPredict HPV Quality Control (QC) real-time PCR assay, employing *CCR5* for molecular cellularity quantification. **Results:** The cellularity values obtained were comparable across the three different methods for all dilutions of the cell line tested. **Conclusions**: The results obtained from this study show that *CCR5* represents a promising molecular marker for the accurate quantification of sample cellularity, confirming its use as a reliable sample adequacy control, thus reducing the risk of “false-negative” results.

## 1. Introduction

The implementation of molecular Human Papilloma Virus (HPV) assays has accelerated programs based on HPV primary screening for the prevention of HPV-related Cervical Cancer (CC). HPV primary screening is a new screening algorithm for women over the age of 30 that involves the use of molecular HPV assays. If the HPV test result is positive, the patient is then referred for cytological testing. The improved clinical sensitivity of molecular HPV assays based on nucleic acid amplification test (NAAT) methods over cervical cytology in screening programs has helped to reduce the incidence and mortality arising from this cancer [1,2,3,4,5]. Moreover, the introduction of molecular HPV assays has allowed the possible transition to the use of HPV primary screening on self-collected samples (vaginal or urine samples autonomously collected by the patient), thereby broadening women’s participation in cervical cancer screening programs.

Self-collected sampling has been widely accepted as a method of cervical screening, especially in low-income and middle-income countries [6,7,8,9,10]. Its acceptance is largely due to its convenience and flexibility in implementation, as it can be performed in various settings such as health care facilities, homes or workplaces facilitated by community health outreach teams. Additionally, HPV molecular assays can be used on self-collected samples as point-of-care cervical cancer screening [7,11,12,13,14,15,16,17,18,19,20,21,22]. Validation of HPV assays on self-samples is therefore becoming increasingly important and essential to provide assurance for their use in routine clinical settings.

The internationally recognized criteria for the validation of HPV tests to be used in cervical cancer screening programs have focused on the importance of assuring the clinical sensitivity and specificity of the assays for the detection of women with precancerous lesions, but less attention has been placed on sample adequacy assessment [5].

Currently, there are several commercially available HPV kits in the global market, but not all of them have been validated according to the international validation criteria, and a recent review by Poljak et al. showed that only about 21% of these kits meet the validation criteria [23]. Furthermore, a systemic review by Arbyn et al. reported that not all validated HPV assays contain an internal control (IC) [24]. In the majority of assays which include an IC, this is associated with the qualitative detection of a *Beta-globin* gene target, used to assess both the presence of human cells in the sample as well as any potential PCR inhibition in the same reaction well used for the detection of HPV DNA targets.

As previously reported, the inclusion of a sample adequacy control (SAC) in a separate reaction well from the molecular diagnostic targets allows a more reliable assessment of sample cellularity, avoiding potential competition for the reaction reagents [25,26,27]. Moreover, a SAC would represent an important quality assurance tool in HPV molecular testing, boosting confidence in negative results [25,28,29]. In particular, the inclusion of a reliable sample adequacy assessment would help to reduce the potential risk of false-negative results due to inadequate sample collection, particularly in cervical screening programs based on self-collected samples, as clearly pointed out in a recent report [5,26].

The importance of evaluating cell counts in other diagnostic laboratory investigations by means of quantitative tools has previously been established and adopted for diagnostic and prognostic analysis in illness such as leukemia and lymphoma [30,31].

The *C-C Motif Chemokine Receptor 5* (*CCR5*) gene has been employed in the OncoPredict HPV assays (Hiantis Srl, Milan, Italy) as an independent Quality Control (QC) reaction to assess sample adequacy through the quantitative determination of the single-copy human *CCR5* gene, also allowing a more reliable HPV viral load determination by normalization according to sample cellularity [32,33]. Similarly, the use of the *CCR5* gene target has also been previously described for the normalization of HIV-1 pro-viral loads in clinical samples [34,35].

This study aimed to evaluate the performance of the molecular target *CCR5* included in the OncoPredict HPV QC module for the reliable quantitation of cell numbers by comparing the results with a cellularity assessment based on a Thoma cell-counting chamber and Fluorescence-activated Cell Sorting (FACS).

## 2. Materials and Methods

**Figure 1:**
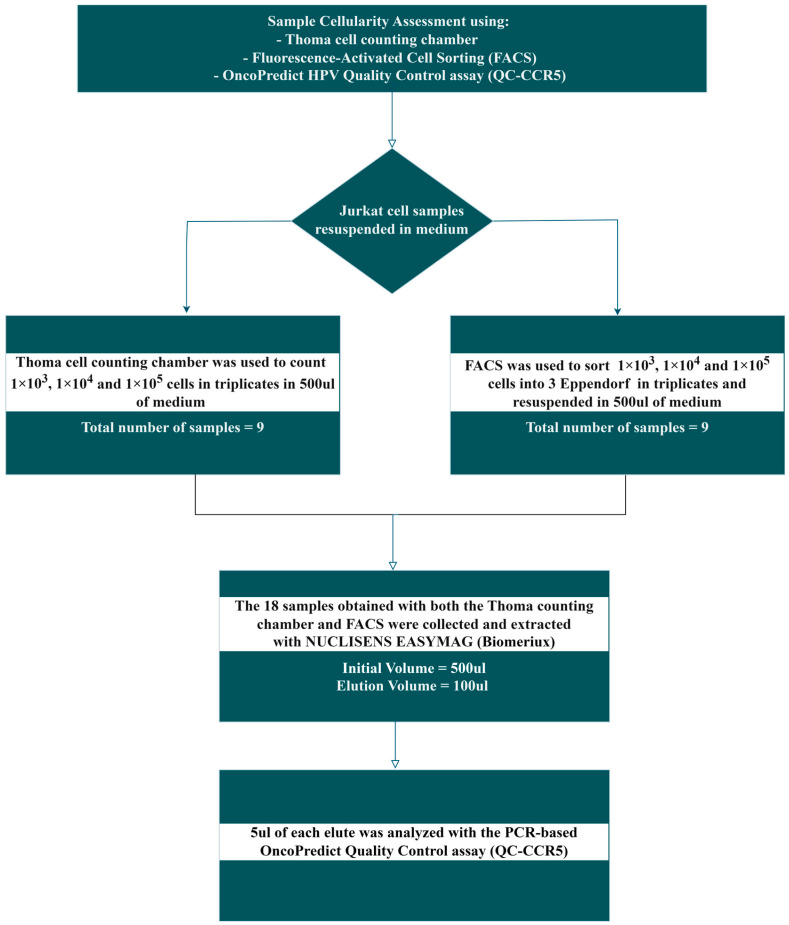
Study workflow.

Human Jurkat cell lines were used to evaluate the accuracy of molecular quantitative cellularity assessment using *CCR5*, a single-copy human gene located on chromosome 3, included as an independent quantitative Real Time PCR (qRT-PCR) reaction of the OncoPredict HPV kit (Hiantis Srl Milan, Italy), by comparing the results with those obtained using two other cell-counting methods based on microscopy cell-counting using a Thoma chamber and Fluorescence-activated Cell Sorting (FACS), as described in the workflow reported in Figure 1.

The frequency of copy number variants and structural variants present in the human *CCR5* gene were also investigated as part of this study to ensure that this gene target could represent a reliable marker in cellularity assessment.

### 2.1. Analysis of the Frequency of Copy Number Variants and Structural Variants in the Human CCR5 Gene

It has been shown that presence of copy number variants (CNVs) or structural variants (SVs) can affect the results of PCR analysis [36]. We thus interrogated a catalog of genomic variants in the Jurkat cell line [37]. We confirmed that *CCR5* is a single-copy gene in these cells and that no copy number variants (CNVs) or structural variants (SVs) affect its coding sequence.

We also explored the frequency in the human population of CNVs and SVs that involve the *CCR5* gene. To this aim, we interrogated the Genome Aggregation Database (gnomAD, v4 release) [38]. GnomAD v4 includes data from ~800,000 individuals. Although the majority of such individuals are of European ancestry, the resource is representative of worldwide genetic diversity, with more than 150,000 samples from subjects with non-European origin (https://gnomad.broadinstitute.org/ accessed on 25 September 2024).

### 2.2. Cell Line: Cell Count with Thoma Chamber

Human Jurkat cell line sample obtained from ATCC (Manassas, VA, USA) was cultured in RPMI-1640 medium supplemented with 10% FBS, 1% Pen/Strep, Fungizone and Glutamine (Complete medium; Euroclone, Milan, Italy) at 37 °C and 5% CO_2_.

After harvesting the Jurkat cells were centrifuged at 1200 rpm for 5 min at room temperature and the pellet was resuspended in 5 mL of complete medium. The cells were then filtered with 40 µm cell strainer (Euroclone) to eliminate cell aggregates.

Cell counting was performed using Trypan Blue solution (Sigma-Aldrich, St. Louis, MO, USA) to exclude non-viable cells, with living cells counted in a double-grid Thoma chamber (Marienfeld Superior, Lauda-Konigshofen, Germany). The Thoma chamber slide was observed using an inverted microscope (Olympus) with a 10× objective. Subsequently, the following cell suspensions of 1000, 10,000 and 100,000 cells were prepared in triplicate in 500 µL (total number = 9 Eppendorf tubes) of complete medium. The remaining cells were used for the cell-sorting procedure.

### 2.3. Cell Line: Fluorescence-Activated Cell Sorting (FACS)

**Figure 2:**
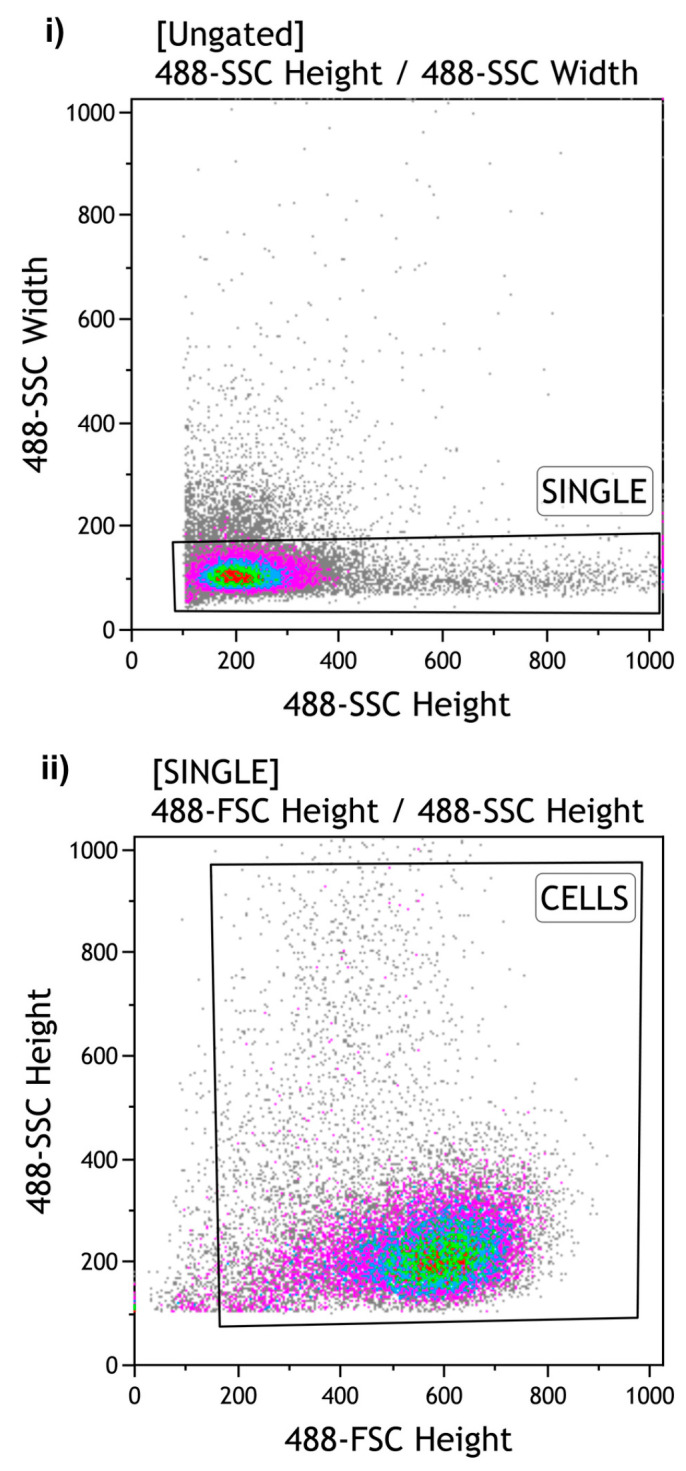
Gating strategy of Flow Cytometry: The gating strategy of Flow Cytometry for the sorting of Jurkat cells was: (**i**) “SINGLE” cells to exclude aggregates (**ii**) “CELLS” to sort the desired cell number. SSC: Side Scatter. FSC: Forward Scatter.

The remaining Jurkat cell suspensions were sorted using a MoFlo Astrios Cell Sorter (Beckman Coulter, Brea, CA, USA). During the Set Sort Decision phase, the following criteria were used: “*Purify and 1-2 Drop*”. Using the “*Limit Event*” function, 1,000, 10,000 and 100,000 cells were sorted in triplicate (Total number = 9 Eppendorf tubes) according to the gate strategy shown in Figure 2. An averaged sort rate of 200–2000 events per second and a sorting pressure of 25 PSI with a 100 µm nozzle were maintained throughout the process. Kaluza software version 1.2 was used for *.fcs* file analysis.

### 2.4. Extraction of Human Jurkat Cell Line Samples

A total of 18 Eppendorf tubes containing the human Jurkat cell line samples obtained following both cell-counting by Thoma chamber and sorting by FACS in a volume of about 500 µL underwent nucleic acid extraction using the automated NucliSENS^®^ easyMag^®^ platform (bioMérieux, Marcy-l’Étoile, France) which exploits BOOM technology with magnetic silica particles diluted 1:2. Prior to extraction, 10 µL of the exogenous spike-in control gene target (synthetic gene-firefly luciferase DNA) included in the OncoPredict HPV Quantitative Typing (QT) kit, used to assess efficiency of nucleic acid extraction as part of the assay’s QC [36], was added to the Jurkat cell line samples. The extraction protocol “Specific B Protocol 2.0.1”, characterized by an elution temperature of 70 °C, was selected and performed according to manufacturer’s instructions using a final elution volume of 100 µL.

### 2.5. Testing of the Jurkat Cell Line Samples with OncoPredict HPV QC Module

The real-time assay of the Quality Control (QC) module of OncoPredict HPV Quantitative Typing (QT) kit (Hiantis SRL, Milan, Italy) was performed on the CFX96™ Real-Time PCR Detection System (Bio-Rad, Hercules, CA, USA) using 5 µL of extracted sample in a total reaction volume of 15 µL. The QC module includes an accurate evaluation of the number of human cells present in the sample through the quantitative determination of *CCR5* gene; the efficiency of nucleic acid extraction through the recovery of an exogenous control gene target added to the sample before preanalytical processing; and the potential PCR inhibition through the amplification of a control target (synthetic custom-designed gene/not human DNA) included in the OncoPredict HPV QT. Standard curves for the quantification of the *CCR5* gene were constructed based on the cycle threshold (Ct) values of 4 quantitative calibrators run in triplicate.

### 2.6. Statistical Analysis

The cellularity was extrapolated from the *CCR5* standard curve and a general estimate of the cellularity value for samples was calculated using the equation below. The *p*-value was calculated using *t*-test for independent samples.

The cellularity value for samples was calculated using Equation (1).

(1) $$=Number\;of\;CCR5\;(cells/reactions)*20\;(\frac{100\;µL\;\left(elution\;volume\right)}{5\;µL\;(innoculated\;volume)})$$

Equation used to determine the sample cellularity value.

## 3. Results

### 3.1. Frequency of CNVs and SVs in the Human CCR5 Gene

Upon interrogation of the Genome Aggregation Database (gnomAD, v4 release) [38], we detected six CNVs and nine SVs that involve the human *CCR5* gene. However, the reported frequency of individual CNVs and SVs was found to be extremely low (the most common one having a frequency of 0.0033), suggesting that they do not pose a problem in the use of this gene as a sample adequacy control.

### 3.2. Performance of CCR5

**Figure 3:**
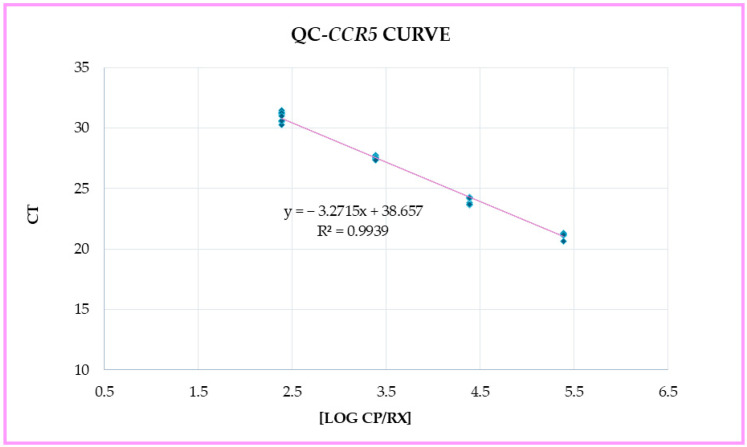
*CCR5* standard curve plot.

After extracting and performing real-time PCR analysis on the 18 Jurkat cell line samples obtained from both the Thoma counting chamber and FACS, the Ct values obtained from each tested sample were used to calculate the number of cells from the *CCR5* standard curve (Figure 3). These cell numbers were reported as *CCR5* cellularity (cells/sample) and Log10 *CCR5* cellularity (cells/sample).

**Figure 4:**
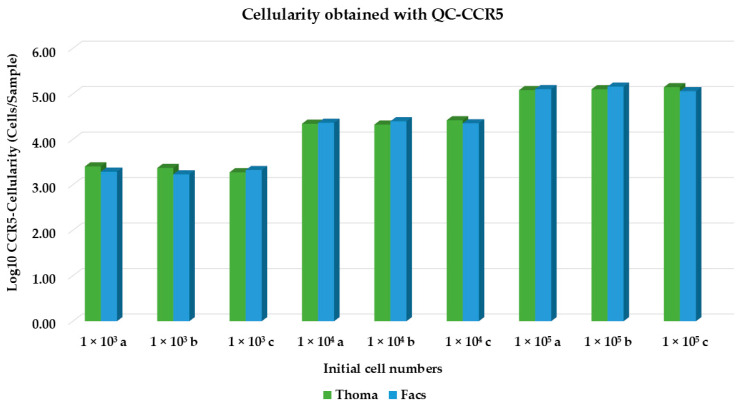
Graphic representation of the QC *CCR5* cellularity value. Cellularity values (Log10 *CCR5* cellularity, cells/sample) are presented for three replicates (a, b, c) across initial cell numbers (10^3^, 10^4^ and 10^5^).

From Table 1 and Figure 4, we observed that the number of cells obtained using the QC-*CCR5* assay correlates to the actual number of cells counted using the Thoma chamber and FACS.

Finally, no significant difference was observed between samples obtained from the Thoma chamber and FACS analysis (*p* = 0.96) using a *t*-test for independent samples.

## 4. Discussion

The diagnostic accuracy of HPV molecular assays has received an important focus in the scientific community, which has led to a consensus on international validation guidelines, known as the “Meijer criteria”, which establish minimum requirements for novel HPV tests in terms of clinical sensitivity, specificity and reproducibility [39]. Additionally, two complementary international initiatives, VALGENT (VALidation of HPV GENotyping Tests) and VALHUDES (VALidation of HUman papillomavirus assays and collection DEvices for HPV testing on Self-samples and urine samples), have been introduced to evaluate the clinical performance validation of HPV assays on cervical and self-collected samples [40,41]. Notwithstanding these validation criteria, only a limited number of studies have focused on evaluating the importance of sample adequacy assessment in molecular HPV-based primary testing in cervical cancer screening [5,25,42]. Moreover, the causes of “invalid results” in molecular HPV testing, which could result from multiple pre-analytical and analytical issues, such as inadequate sampling, sample processing and/or PCR inhibition, have not been fully investigated. Furthermore, presently commercially available HPV assays which include an internal human gene control often apply arbitrarily chosen IC cut-offs, in terms of cycle threshold (Ct) values, by the assay manufacturers, which have not undergone more stringent validation, such as those that are presently required for determining HPV cut-offs in terms of clinical sensitivity and specificity. To our knowledge there are no previously reported studies evaluating what should be considered an appropriate number of human cells in relation to sample adequacy for molecular HPV testing.

The results of our study, comparing the performance of molecular *CCR5* detection to microscopy and FACS cell-counting, showed that the cellularity assessment was comparable between the different methods.

Our study results for using *CCR5* to assess cell counts align with the study conducted by Malnati and his group. In their study, *CCR5* as a single-copy human gene was used to determine the cellularity of human cells from clinical samples to achieve the absolute quantification of proviral HIV-1 DNA [34]. Similarly, a study carried out by Hong et al. used *CCR5* copy numbers to calculate HIV-1 DNA copies per 1 million cells [35]. Additionally, Cocuzza et al. reported the use of *CCR5* to determine the number of human cells present in clinical samples, thereby enabling the normalization of viral copy numbers per cell for the accurate quantification of HPV viral loads. This highlights an additional advantage of using *CCR5* [33].

The presence of a sample adequacy control (SAC) in HPV assays is also expected to ensure the reliability of negative results by confirming the quality of the clinical sample to be tested and of the sample-collection procedure. In cervical cancer screening molecular HPV testing, a “fixed” volume of resuspended cervical or self-sample is used to perform the analysis based on manufacturers indications, irrespective of the abundance of sample collection. The latter is influenced by the fact that both clinician-collected cervical samples and self-collected vaginal samples originate from the brushing or swabbing of a mucosal surface which is influenced by the sampling procedure and by the person who is performing the collection, as also previously reported for the mucosal sampling used for SARS-CoV-2 molecular testing [43]. Moreover, negative results from molecular tests may not only be due to the absence of cells but also to errors in extraction or amplification processes [26]. The OncoPredict HPV QC test addresses these issues by including an exogenous control for both nucleic acid extraction and PCR amplification, which enable the separate monitoring of both the extraction efficiency and the potential PCR inhibition for each sample.

A SAC might be external, as in the case of the OncoPredict HPV assay, where it is amplified in a separate reaction well, or as an internal control (IC), where it is co-amplified with HPV nucleic acid [25]. Nevertheless, an appropriate IC molecular assay cut-off needs to be established in order to appropriately assess the sample adequacy.

Although no cost–benefit analysis has been performed, the additional detection of a reliable SAC, especially if it is performed in a separated reaction, is of course associated with an increased cost of the test which can however be justified by more reliable and robust results.

The reliability of negative results in HPV screening is critical for patient health, as it can influence clinical decisions. HPV testing, with its strong negative predictive value, has allowed the extension of screening intervals from approximately three years with cytology-based primary-screening protocols to five years for those who test negative [29]. This could mean that an undetected infection or early-stage precancerous lesions could progress into more advanced disease, potentially resulting in cervical cancer due to untimely follow-up care.

The risk of inadequate samples being collected is expected to increase with the recent move promoted by the World Health Organization (WHO) towards the use of self-collection for cervical cancer prevention, which includes vaginal samples being collected by the woman herself. Additionally, inadequate samples may also represent a problem in clinician-collected cervical samples, particularly in relation to women’s age. It has been shown, for example, that in post-menopausal women the transformation zone retreats further into the cervical canal, making it more difficult to obtain adequate samples. All these factors can impact the quality assurance of molecular HPV testing of clinical samples [44,45,46].

One of the major drawbacks of the use of molecular HPV assays is the lack of standardized criteria regarding the minimum cellularity required to define sample adequacy. The use of molecular HPV assay across various clinical settings requires the implementation of the quality assurance parameters stated by Cuschieri and colleagues [5].

On the contrary, parameters have been well-defined for both traditional Pap smears and for cervical liquid-based cytology, with a required minimum cellularity count of 8000 cells and 5000 cells, respectively, according to the Bethesda system [47]. A better understanding and further studies are needed to assess quality assurance in the performance of the more recently introduced HPV-based primary cervical cancer screening programs. In particular the assessment of the minimum cellularity required to define molecular cut-offs for sample adequacy it is necessary by the use of appropriate human gene target with a reliable number of copies per cell.

To the best of our knowledge, this is the first study comparing the performance of *CCR5*, a single-copy human gene target, to other laboratory reference cell-counting methods for the accurate molecular assessment of human cell numbers. The limitations of this study include the small number of cell-line samples tested and that these were not derived from true clinical samples, nor human vaginal or cervical cells. Moreover, this study did not include a direct comparison with other human genes such as *beta-globin* (*HBB*), ARHGEF11, *DNA topoisomerase III* (*TOP3*) and other available internal controls already included as SAC in presently available commercial HPV assays. Future studies will need to include a larger number of clinical samples allowing to better evaluate the range of cellularity required for quality assurance in molecular HPV testing and a direct comparison of *CCR5* gene to other available internal controls [42].

## 5. Conclusions

The results obtained from this study show that *CCR5* represents a promising molecular marker to assess sample cellularity in molecular diagnostics, with particular reference to molecular HPV testing used in cervical cancer screening. It will be important in future to define specific cellularity cut-off values for specific sample types and for the quality assurance of molecular HPV-based primary screening through the use of samples collected from human mucosal surfaces.

## Figures and Tables

**Table 1:** Cellularity values obtained from the analysis of QC *CCR5* assay across three different initial cell numbers (10^3^, 10^4^ and 10^5^). Each initial cell number had three replicates (a, b, c). * (Initial cell numbers indicate the absolute cell count (1,000; 10,000; 100,000) detected through both the Thoma chamber and FACS methods.)

	Triplicate Samples Counted Using Thoma Chamber	Triplicate Samples Counted Using FACS
Initial Cell Number *	*CCR5* Molecular Cellularity Determination (Cells/Samples)	
**10^3^ a**	2.54 × 10^3^	1.94 × 10^3^
**10^3^ b**	2.35 × 10^3^	1.70 × 10^3^
**10^3^ c**	1.89 × 10^3^	2.11 × 10^3^
**10^4^ a**	2.20 × 10^4^	2.31 × 10^4^
**10^4^ b**	2.11 × 10^4^	2.50 × 10^4^
**10^4^ c**	2.63 × 10^4^	2.25 × 10^4^
**10^5^ a**	1.21 × 10^5^	1.27 × 10^5^
**10^5^ b**	1.26 × 10^5^	1.44 × 10^5^
**10^5^ c**	1.40 × 10^5^	1.14 × 10^5^

## Data Availability

Final datasets generated by the study are stored locally and securely at the University of Milano-Bicocca and will be available by request to the corresponding author.
